# Medical abortion kit dispensing practices of community pharmacies in Pokhara Metropolitan, Nepal

**DOI:** 10.1371/journal.pone.0244969

**Published:** 2021-01-13

**Authors:** Nim Bahadur Dangi, Sangam Subedi, Mahasagar Gyawali, Aashish Bhattarai, Tulsi Ram Bhandari

**Affiliations:** 1 Faculty of Health Sciences, School of Health and Allied Sciences, Pokhara University, Pokhara, Nepal; 2 Janamaitri Foundation Institute of Health Sciences (JFIHS), Hattiban, Lalitpur, Nepal; College of Pharmacy & Health Sciences, UNITED STATES

## Abstract

**Background:**

Medical abortion (MA) refers to the use of medicines to terminate the pregnancy. There is an urgent need to spread safe abortion services in the community. This study assessed the MA kit dispensing practices of community pharmacies in Pokhara Valley, Nepal.

**Methods:**

A cross-sectional descriptive study was conducted in Pokhara Valley, Nepal from December 2017 to November 2018. Among the community pharmacies of Pokhara Valley, 115 community pharmacies were selected using a consecutive sampling method. A semi-structured questionnaire was used to collect data. MA kit and related information were requested by simulated male clients visiting the community pharmacies. The information obtained from the pharmacy workers was recorded in the data collection sheet.

**Results:**

Nine brands of MA kit from eight manufacturing companies were found in practice in Pokhara Valley, out of those only five (56%) were registered in Nepal. Seven brands were sold at more than the labeled price. The pharmacy workers asked about the gestational age and confirmation of pregnancy in all the cases. Most of them counseled the clients on the frequency, duration, and direction of use. Dispensing practice and level of counseling were found to be significantly correlated (r = 0.40, p value = 0.01).

**Conclusion:**

Despite the awareness of the pharmacy workers on the MA kit, most of them provided limited information to the clients. Nearly half unregistered MA kits were found in practice at the community pharmacies. Thus, the Department of Drugs Administration and other concerned authorities must provide relevant training and awareness programs to the pharmacy workers of the community pharmacies for preventing the malpractice of MA kit. The Government of Nepal must restrict the illegal entry of unregistered brands and assure the standards of MA kit by regulating drug acts and policies effectively.

## Introduction

Abortion refers to the termination of pregnancy before the fetus has attained the viability of independent extra-uterine life. Annual 68,000 women die, and 5.3 million disabilities occur as a sequence of unsafe abortion in lower and middle-income countries [1,2]. Nepal is often considered to be a model of effective execution of safe abortion services after the legalization of abortion [3]. The first-trimester surgical abortion, second-trimester abortion training, and medical abortion started in 2004, 2007, and 2009 respectively even though abortion was legalized in 2002 in Nepal [4]. Abortion is permitted for up to 12 weeks of the gestation on demand and up to 18 weeks of the gestation in case of rape or incest and at any stage on the recommendation of the doctor if the pregnancy causes any harm to the life of the women or the women have any physical or mental health problem or if any deformities in the fetus [5]. Despite the legalization of abortion, a large number of illegal abortions were practiced in Nepal [6,7]. Medical Abortion (MA) is accessed through an accredited safe abortion service and as well as through pharmacies in Nepal. The occurrence of barriers for safe abortion services led to a demand for medical abortion through community pharmacies. This indicated need for the spreading of safe abortion services in community pharmacies [8,9].

MA kit, a combination of either mifepristone and misoprostol or misoprostol only, has contributed to task shifting and sharing. This has reduced the need for skilled surgical abortion providers [10]. MA kit has been accepted as an effective and safe option for early abortion [11–13]. Among 98,640 safe abortion service users, 62% terminated pregnancy by MA in 2017/18 [14,15]. The preference of pregnant women for MA over Manual Vacuum Aspiration (MVA) is related to the non-invasive nature of MA and the internal examination required in MVA that causes hesitation among women [16]. The preference of MA by a pregnant woman is higher in countries where the health system is fully supportive or completely disengaged over surgical abortions [17].

Ministry of Health and Population of Nepal through national guidelines of 2009 allowed the use of MA up to nine weeks of gestation. MA kits at community pharmacies are not allowed to be sold as the counter product (over the counter product) as per the drug act of Nepal. Despite legal restrictions, various brands of MA kits are available at community pharmacies and sold to clients without prescription [18]. In countries like Nepal, community pharmacies act as the first contact point for abortion services. Registered and unregistered MA kits are easily available at community pharmacies in various brands [19].

Nepal is a country of cultural diversity. The majority of communities in Nepal are patriarchal. Patriarchy is the struggle between women and men to control women's labor power and most of the women's life is influenced by her father and husband [20,21]. Community pharmacies offer more privacy and convenience than offered by accredited clinical facilities [22]. So, it is easier to visit pharmacies for MA kits and other reproductive health matters to husbands/partners compared to public health facilities.

Various studies have discussed issues of the availability of unregistered MA kits [19,23]. Medicine registration is mandatory for marketing authorization and an important domain under better-quality medicines. Registered medicines perform better than unregistered medicines [24,25]. Pharmacists, assistant pharmacists, and professionals are legally qualified to dispense any medicaments from the pharmacy after a prescription from medical doctors [26]. There are many community pharmacies there are no legally qualified pharmacy workers. No specific training is provided on an MA in Nepal. World Health Organization (WHO) estimated that more than half of all medicines are inappropriately prescribed, dispensed, or sold in the world [27]. Pharmacy workers can dispense only the prescribed medicines (except over the counter drugs) but in practice, most of the community pharmacies dispense without prescription in Nepal [28]. Many of the studies have recommended a trained pharmacy worker for effective dispensing of MA kit with an assessment of knowledge regarding MA kit and proper counseling [19,29]. So, this study assessed the status of registration, manufacturer, and average price for MA kits in Nepal, dispensing and counseling practice of pharmacy workers, and the relationship between dispensing and counseling practice of the pharmacy workers.

## Methods

This is a cross-sectional descriptive study conducted in Pokhara Valley, the capital city of Gandaki Province of Nepal from December 2017 to November 2018. A total of 115 community pharmacies* were selected using a consecutive sampling method [30]. Among 413 registered community pharmacies in Pokhara valley, one in four pharmacies, as they came across, were visited by simulated clients leading to 115 pharmacies [31,32]. This study was conducted after receiving formal permission from the Department of Drug Administration (DDA), and ethical approval from the Institutional Review Committee (IRC), Research Center, Pokhara University, Nepal (ref. no. 108/074/75). National Health Research Council (NHRC), the apical body for all health research in Nepal, is allowed to publish ethical guidelines as per the Nepal Health Research Council Act 1991. National Ethical Guidelines for Health Research in Nepal, 2011 does not provide distinct guidelines for simulated client study. IRC of Pokhara University is affiliated with the National Ethical Review Board of NHRC. The data were collected using a semi-structured questionnaire with a simulated client (SC) approach. The questionnaire was prepared based on literature review by the researchers. The preliminary draft of the research tool was pretested and the reliability and validity of the tool were ensured. SC approach has been used for more than two decades in health care research [7]. In reproductive health studies, the SC approach has been commonly used in various studies related to information provided by pharmacy workers** [8,33,34].

Ten males were selected and trained regarding abortion, MA kit, questionnaire, and questioning techniques. These males were considered trained male simulated clients. They were trained to act as the husband of a pregnant woman.

The SC requested the pharmacy workers to give MA kit and its' information. SC visited community pharmacies without prescription as assessing different brands and their registration status and price variation were aims of the study. The conversation between SC and the pharmacy workers were audio recorded. Manufacturing company, brand name, labeled price, and expiry date of the MA kit were observed by SC. The community pharmacy workers were also asked for further information like a drug dose, route of administration, the direction of use, price, and side effects of medicines. At the end of the interview, the interviewed pharmacy workers were informed of the research and audio recording. Those pharmacy workers who did not give consent were not included in the study and their recordings were immediately deleted. As informed consent was taken after the interview, the bias in results associated with dispensing practice was reduced. The information obtained from the pharmacy workers was recorded in the data collection sheet based on audio recording and observation of the MA kit after leaving community pharmacies.

Dispensing practice and counseling levels were calculated and categorized using the percentile method [35]. For assessing the dispensing practice and counseling level of pharmacy workers, five questions, and thirteen counseling items were set respectively. Five questions set were gestational age, confirmation of pregnancy, frequency of use of MA kit, age of pregnant woman, and permission from family/partner. Thirteen counseling items were frequency and duration of use, the direction of use/route of administration, possible side effects, drug dose, follow up, name and description of the drug, precaution, contraindications, referral, family planning/contraceptives use, other medications being used, special direction and possible drug interaction. Frequency of questions was calculated adding all questions asked and as well as question repeated to SC for assessing the dispensing practice of pharmacy workers whereas for assessing counseling level of pharmacy workers, frequency of counseling item was calculated adding all counseling items counseled as well as several times the same counseling item used for counseling. Hence the chance of score may be from zero to any values. After obtaining the value, zero to that value was categorized into three groups using percentile. It was operationalized up to 33.33% as low, 66.33% as a medium, and remains as good. Those purchased pills were removed from their packs and grounded. The homogenous paste, prepared with water, cement, and lime, set as a solid mass was discarded to prevent the illegal use of medicines and this is the safest way of disposal [36].

* A ground level unit of medicine outlet from where pharmacy services (dispensing of medicines and counseling of patients) are provided.

** The person who dispenses the medication from the pharmacy and is not necessarily a pharmacist or assistant pharmacist or recognized by a regulatory authority for dispensing.

## Results

Out of 115 pharmacy workers, there were 33 (28.70%) females and 82 (71.30%) males. In community pharmacies visited by SC, nine different brands of MA kit were found. The names of brands were replaced with notation A1 to A9 instead of the real brands' name for ethical reasons. Those brands were manufactured by Nepalese (22.22%) and Indian (77.78%) pharmaceutical companies. Among those, brand A1 was dispensed by most community pharmacies. Out of nine available brands, only five were registered (A1, A3, A4, A7, and A8) in DDA, the regulatory authority of medicines in Nepal (Table 1).

**Table 1:** Available brands of medical abortion kits in Pokhara Valley, Nepal.

**S.N**	Available Brands	No. (Percent)	Manufacturer *	Registration status
1	A1	16 (13.91)	IC	Registered
2	A2	15 (13.04)	IC	Unregistered
3	A3	14 (12.17)	NC	Registered
4	A4	14 (12.17)	IC	Registered
5	A5	12 (10.43)	IC	Unregistered
6	A6	11 (9.57)	IC	Unregistered
7	A7	11 (9.57)	IC	Registered
8	A8	11 (9.57)	NC	Registered
9	A9	11 (9.57)	IC	Unregistered
	Total	115 (100)	IC = 7 (77.78%)	Registered = 5 (55.56%)
			NC = 2 (22.22%)	Unregistered = 4 (44.44%)

*IC = Indian Company, NC = Nepalese Company.

The labeled price of nine dispensed brands was listed by observation of the label. The selling price of those brands was listed by asking the price of purchase. The selling prices were different from each other and also from the labeled price in all community pharmacies. Most brands (7 out of 9) were sold at more prices than labeled (Table 2).

**Table 2:** Marked price versus selling price of available brands.

S.N	Brands	Labeled Price ($)	Selling Price ($)		
			Maximum	Minimum	Average
1	A3	4.84	5.27	4.84	4.99
2	A6	4.57	5.27	4.57	4.97
3	A7	5.31	5.27	4.40	4.90
4	A8	5.55	7.03	4.40	5.72
5	A4	3.52	4.57	3.52	3.67
6	A5	5.49	7.03	5.71	6.26
7	A9	4.40	5.27	4.40	4.88
8	A1	9.71	10.55	8.79	9.78
9	A2	5.71	5.27	536	4.97

* **1 $ = 113.75 Nepalese Currency.**

The results show that the gestational age and the confirmation of pregnancy were asked in all the cases whereas questions about frequency of use of abortion kit, age of the user, and permission from/discussion with family or partner were asked by 94.78%, 90.43%, and 86.08% of pharmacy workers respectively (Table 3).

**Table 3:** Questions asked to SC by pharmacy workers before dispensing (n = 115)*.

**S. N.**	**Possible questions**	**No.**	**Percent**
1	Gestational age	115	100
2	Confirmation of pregnancy	115	100
3	Frequency of use of abortion kit	109	94.78
4	Age	104	90.43
5	Permission from/discussion with family or partner	99	86.08

*Multiple responses.

Majority of the pharmacy workers during dispensing counseled on frequency and duration of use (99.13%), the direction of use/route of administration (96.52%) whereas very few pharmacy workers counseled about other medications (38.26%), special direction (32.17%) and drug interaction (26.96%) (Table 4).

**Table 4:** Counseling during dispensing of MA kits (n = 115)*.

S. N.	Counseling	No.	Percent
1	Frequency and duration of use	114	99.13
2	The direction of use/route of administration	111	96.52
3	Possible side effects	109	94.78
4	Doses of drug	106	92.17
5	Follow up	91	79.13
6	Name and description of drugs	95	82.61
7	Precaution	84	73.04
8	Contraindication	70	60.87
9	Referral	67	58.26
10	Family planning/contraceptives used	51	44.35
11	Other medications	44	38.26
12	Special direction	37	32.17
13	Drug interaction	31	26.96

*Multiple responses.

For assessing the dispensing practice and counseling capability of pharmacy workers, we asked five and 13 questions respectively. For assessing dispensing practice the score ranged from three to twelve (value obtained by adding all questions asked and as well as question repeated). Using percentile, we operationalized the score up to five as low (33.33%), six to eight as medium (66.33%), and remains as good. Similarly, for assessing the counseling capability the scores ranged from five to seventeen (adding all counseling items counseled as well as several times the same counseling item used for counseling) and operationalized the score up to eight as low (33.33%), nine to twelve as medium (66.33%), and remains as good. Out of the total involved dispensers, sixty-four, forty-four, and seven pharmacy workers as a low, medium, and a good level of dispensing practice respectively whereas thirty, sixty-three and twenty-two pharmacy workers with a low, medium, and a good level of counseling capabilities respectively. The relationship between dispensing practice and counseling capability was found to be significantly associated (r = 0.40, p = 0.01). The pharmacy workers who had good dispensing practice had also performed good counseling responsively (Table 5).

**Table 5:** The relation between dispensing practice and level of counseling of pharmacy workers.

Level	Dispensing practice		Counseling Capability	
	No.	Percent	No.	Percent
Low	64	55.65	30	26.08
Medium	44	38.27	63	54.78
Good	7	6.08	22	19.14

Pearson correlation (r) = 0.40, p value = 0.01.

## Discussion

This study assessed the dispensing practices of MA kit in community pharmacies in Pokhara Valley, Nepal. More than two-thirds (71.30%) of dispensers were males. A total of nine different brands of MA kit of various manufacturing companies were found, out of the total practiced kits only 55.56% were registered in the DDA. Among nine different dispensed brands, only two brands were Nepalese pharmaceutical companies whereas the remaining seven brands were of Indian pharmaceutical companies. Among those nine brands, only five brands were registered in DDA, the remaining four brands were not registered and those unregistered brands were manufactured by Indian companies. For the prevention of irrational and inappropriate use, medicines are registered in the concerned authorities and made accessible in a country [37]. According to Section 17 of the Drug Act of Nepal, 1978 [26] the pharmacists, assistant pharmacists, and professionals can only dispense medicines. These medicines must be registered in the DDA. But the community pharmacies involved the pharmacists, assistant pharmacists and professionals with legal rights and also involved health workers, nurses and other health workers without legal rights.

The reason for selling unregistered brands may be due to the influence of local marketing agents having different business strategies such as ‘buy one, get one free’ and ‘heavy discount’ in unregistered brands and an open border between Nepal and India [19].

If adequately trained the pharmacy workers with legal rights, they can dispense MA kit safely and effectively with or without prescription. A study conducted in Nepal concluded that the role of the pharmacy workers as a provider of the MA kit needs to be recognized by the government. The government can also formulate the policies to regulate the pharmacy workers and aware the clients providing complete information about the MA kit [38].

Information about the gestational age and pregnancy confirmation was asked to SCs by all the pharmacy workers. This indicates that maximum pharmacy workers were aware to ask about the gestational age and pregnancy confirmation before dispensing the MA kits. The quality of the information provided to client was observed to be low in a survey conducted in Uttar Pradesh, India which highlighted the gaps in knowledge among pharmacists [15] whereas the pharmacy workers had a low level (6.08%) of dispensing practice followed by medium level (38.27%). This result resembles the findings of studies conducted before where inaccurate information provided and ineffective medications found to be dispensed by pharmacists were documented [19,39]. A study in Nepal suggested the involvement of the Government of Nepal in formulating policies that recognize the role played by pharmacy workers in the provision of medicines for safe, early medical abortion and the prevention of unsafe abortions [19].

In a patriarchal society like Nepal, married women are under intense pressure to have a male child that could contribute to abortion [40]. The pressure on women to bear sons as well as knowing those seeking abortion for gender preferences is a difficult task for health workers [41]. The fertility and reproductive behavior of women are affected by sex selection [42]. Most of the pharmacy workers counseled about frequency and duration of use of kit (99.13%), whereas only 26.96% of the pharmacy workers asked about the drugs being used indeed mifepristone is likely to interact with various drugs. Mifepristone is metabolized by the CYP3A4 enzyme so CYP3A4 inducers, for examples rifampicin, barbiturates, carbamazepine, may decrease the effectiveness whereas CYP3A4 inhibitors, for examples ketoconazole, erythromycin, may increase the side effects of mifepristone [43]. Regarding the level of counseling the performance of a maximum number of pharmacy workers was medium. A prospective study conducted in Nepal highlighted that 83.33% pharmacy workers having certain problems during patient counseling even though 56.67% of pharmacy workers knew that the counseling is important during dispensing of medications [44].

This research was conducted in the Pokhara Valley of Nepal where the community pharmacies are very densely available in the urban areas. It was difficult to find the sample considering the urban vs rural pharmacies, chain/group practices vs. independent pharmacies, pharmacist vs. pharmacy assistant, etc. Most of the pharmacies from the urban areas of the Pokhara Valley were selected. Pharmacy workers were promised while taking consent so the brand name of MA kits was blinded. Dispensing unregistered products and as well as dispensing MA kit without prescription of legalized person, this information regarding pharmacy workers could not be taken. Male clients only were used as female clients feel shy to express their pretended pregnancy. If female clients could have been used the difference in counseling of male and female clients could be assessed.

## Conclusions

The unregistered MA kits were found in practice in Nepal which were manufactured and supplied by Indian Pharmaceutical Companies. This availability of unregistered MA kit may be due to the porous border between Nepal and India. The government of Nepal must restrict the illegal entry of the unregistered brands and assure the standards of MA kits in Nepal by regulating drug acts and policies effectively. Most of the pharmacy workers counseled their patients with minimum information. Safe abortion outcome is the most wanted which can be met with training pharmacy workers in harm reduction strategies that could improve dispensing practice among pharmacy workers leading to improve counseling. Safe abortion is a great matter of concern for reproductive health in reducing maternal mortality. So, pharmacy workers should train regarding reproductive rights and decision-making on reproductive matters. Pharmacy workers should consider the acceptance of abortion and explain the merits and demerits of the use of MA kits to women precisely. Thus, concerned authorities should focus to produce competent pharmacy workers with sound dispensing practice of counseling skills and, provide relevant training and awareness programs regarding selling and counseling of the MA kits.

## Supporting information

S1 Dataset Minimal data set. (XLSX)
